# Osmotic pressure-adaptive responses in the eye tissues of rainbow smelt (*Osmerus mordax*)

**Published:** 2011-10-05

**Authors:** Robert L. Gendron, Elizabeth Armstrong, Hélène Paradis, Lacey Haines, Mariève Desjardins, Connie E. Short, Kathy A. Clow, William R. Driedzic

**Affiliations:** 1 Division of BioMedical Sciences, Faculty of Medicine, Memorial University, St. John’s, NL, A1B 3V6, Canada; 2 School of Optometry, University of Waterloo, Waterloo, ON, Canada; 3 Ocean Sciences Centre, Memorial University of Newfoundland, St. John’s, NL, Canada

## Abstract

**Purpose:**

The rainbow smelt (*Osmerus mordax*), is a teleost fish, which avoids freezing by becoming virtually isosmotic with seawater. The effects that such massive changes in osmolarity have on both its visual system and its highly evolved and specialized circulation are not known. New knowledge about the osmotic adaptation of the rainbow smelt eye is highly relevant to the adaptation and survival of this species and to its ability to feed as a visual predator in the face of environmental pressures. Moreover, the molecular physiologic response of the smelt to osmotic stress might provide valuable insights into understanding and managing mammalian pathological hyperosmolarity conditions, such as diabetes. We undertook the present study to provide an initial assessment of gene expression in ocular vasculature during osmotic adaptation in rainbow smelt.

**Methods:**

Immunohistochemistry with species cross reactive antibodies was used to assess blood vessel protein expression in paraffin sections. Western blotting was used to further verify antibody specificity for orthologs of mammalian blood vessel proteins in rainbow smelt. Thermal hysteresis and the analysis of glycerol concentrations in vitreous fluid were used to assess the physiologic adaptive properties of cold stressed eyes.

**Results:**

Glycerol levels and osmotic pressure were significantly increased in the vitreal fluid of smelt maintained at <0.5 °C versus those maintained at 8–10 °C. Compared to the 8–10 °C adapted specimens, the rete mirabile blood vessels and connecting regions of the endothelial linings of the choroidal vessels of the <0.5 °C adapted specimens showed a higher expression level of Tubedown (Tbdn) protein, a marker of the endothelial transcellular permeability pathway. Expression of the zonula occludens protein ZO-1, a marker of the endothelial paracellular permeability pathway showed a reciprocal expression pattern and was downregulated in rete mirabile blood vessels and connecting regions in the endothelial linings of choroidal vessels in <0.5 °C adapted specimens. Smelt orthologs of the mammalian Tbdn and zoluna occludens protein 1 (ZO-1) proteins were also detected by western blotting using anti-mammalian antibodies raised against the same epitopes as those used for immunohistochemistry.

**Conclusions:**

This work provides the first evidence that molecules known to play a role in ocular vascular homeostasis are expressed and may be differentially regulated during anti-freezing cold adaptation in smelt eyes. We propose a hypothesis that in a state of cold-induced hyperosmolarity, changes in ZO-1 expression are associated with the passage of small solutes from the plasma space to ocular fluid, while changes in Tbdn expression regulate the passage of proteins between the ocular fluid and plasma space. This work also provides fundamental insight into the mechanisms underlying the adaptation of the blood-retinal barrier to metabolically relevant compounds such as glycerol.

## Introduction

In the absence of protective mechanisms, the body fluids of a teleost fish should, based on osmotic pressure alone, freeze at about −0.65 °C. The rainbow smelt, a species common to the North Atlantic, avoids freezing in winter temperatures as low as the freezing point of seawater (−1.8 °C), through both non-colligative and colligative means. This species, in common with many other low temperature-adapted marine fish, produces a cysteine-rich, type II anti-freeze protein, albeit at low levels [1], but is unusual among fish in that it also accumulates glycerol, urea, and trimethylamine oxide in the plasma and other tissues (muscle, heart, liver, spleen, kidney, gill, intestine, and brain), such that osmotic pressure increases from about 325 up to 1,000 mOsmols [2–4]. In effect, the fish approaches an isosmotic state with seawater. To maintain glycerol levels, smelt feed upon crustaceans and other invertebrates at low temperatures, implying that their visual system remains functional.

The fish eye is dependent upon a highly evolved and specialized circulation [5–7]. In teleost fish, retinal tissues are supported by vascular structures-the fenestrated choroidal vasculature (also called the choriocapillaris) and the non-fenestrated inner retinal vasculature-which are largely similar to those occurring in mammalian retinas [8]. However, unlike mammals, teleosts also have a rete mirabile, a vascular organ that is positioned just posterior to and upstream of the choroidal vasculature. The rete mirabile concentrates oxygen for delivery to the neural retinal tissues via the choroidal and retinal vasculatures [5,6].

In mammals, much of the retina exists behind a blood-retinal barrier and several proteins appear to be particularly important in the trafficking of solutes and proteins from the plasma space, across the vascular membranes, and into the retinal layers of the eye. Tubedown (Tbdn) and zoluna occludens protein 1 (ZO-1) are two such proteins, which are, respectively, associated with the transcellular and paracellular permeability pathways of the blood vessels. Our laboratory initially characterized and has described the importance of expression of the N-terminal acetyltransferase subunit Tbdn (also now known as Naa15) in choroid-retinal blood vessels to maintain homeostasis of the mammalian retina [9–12]. Knockdown of Tbdn in animal models and the suppression of Tbdn expression in human retinal disease specimens are associated with the loss of retinal integrity [13]. Tbdn is evolutionarily conserved, associates with the actin binding protein Cortactin, and is involved in the regulation of retinal endothelial cell transcellular permeability to Albumin [14]. ZO-1 is a membrane-associated guanylate kinase homolog, which interacts with both Cortactin and F-actin, and which is essential to the assembly of tight junctions [15,16]. Two unique motifs in ZO-1 regulate the tight junction-specific localization and organization of transmembrane proteins that are necessary for the formation of the paracellular barrier [15]. One of these motifs corresponds to the F-actin binding domain of ZO-1 [16]. The mechanism of ZO-1 expression in a teleost has recently been described [17].

Smelt, unlike other fish species, display dramatic changes in plasma osmolarity. Massive changes in osmolarity present in the sub-zero marine environment might affect the ability of smelt to see, swim, and function since the eye interfaces directly with seawater. However, such physiologic adaptation to freezing does not seem to disturb efficient vision, since smelt rely on visual identification in the pursuit of prey, regardless of the water temperature [18]. Nothing is known about gene expression in the retinal vasculature of smelt or about how molecular expression patterns might change in smelt retina during physiologic adaptation to cold and osmotic stress. Although several studies address the impact of low temperatures on a fish lens [19,20], we know of only one investigation related to the freeze resistance of ocular fluids. Turner et al. [21] concluded that several Antarctic fish, living at −1.9 °C, depress the freezing point of ocular fluids using supercooling. In these species, the osmotic pressure of both the aqueous and vitreous humors was lower than in serum, and was only about half that of seawater. Moreover, only trace amounts of glycopeptide antifreeze were observed in ocular fluids despite the high levels present in serum. New knowledge about the ability of the smelt retina to adapt to temperature and osmotic stress is highly relevant to the adaptation and survival of this species in the face of environmental pressures such as climate change. Moreover, this species may serve as a unique and valuable model system for studying retinal responses to changing osmotic environments. In this study, we assess whether such changes are associated with freeze resistance in the vitreous humor given the intimate connection between the plasma and ocular fluids via the rete mirabile, and the elevated levels of small metabolites in the plasma during winter. Although the choriocapillaris appears to be histologically similar in both smelt and mammals, we hypothesized that functional differences might exist in smelt due to the presence of the rete mirabile and specialized mechanisms associated with the maintenance of the visual system under freezing conditions.

## Methods

Rainbow smelt (*Osmerus mordax*) were captured by seine netting at Mount Arlington Heights, Placentia Bay, Newfoundland, Canada, from late October to mid-November in the years 2007, 2008, and 2009. The smelt were transported to the Ocean Sciences Centre at the Memorial University of Newfoundland. The fish were maintained in seawater, on a natural photoperiod with fluorescent lights set on an outdoor photocell, and were fed chopped herring twice a week to satiation. Upon arrival at the laboratory, the smelt were randomly sorted into tanks that received either seawater heated to 8–10 °C (which is similar to the average ocean temperature at the time of capture [22]) for the duration of the experiments, or water that tracked ambient temperature and that decreased to less than 0.5 °C in February and March. The time lapse from collection of the fish to experimentation was between five and six months. All fish appeared to be in healthy condition and well fed. As such, there was no reason to believe that the fish were unduly stressed. Fish held at 8–10 °C are hereafter referred to as “warm fish,” while the fish held in ambient water are referred to as “cold fish.” A typical temperature profile is presented in Lewis et al. [22]. The fish were sampled in March 2007 to determine the glycerol level in the vitreous humor, and were sampled again in February and March 2009 and 2010, respectively, to determine plasma glycerol levels, as well as the glycerol level, osmotic pressure, and thermal hysteresis in the vitreous humor. Fish from the 2007, 2009, and 2010 samplings were used in immunohistochemical analyses and western blot analyses of the ocular tissue. The care, dispatching, and use of animals in this study followed the guidelines set forward by the Canadian Council on Animal Care and were approved by the Institutional Animal Care Committee of Memorial University.

Blood was collected in heparanized syringes via caudal puncture, was centrifuged for 5 min at 5,000× g, and the plasma was removed and stored at −80 °C. The fish were sacrified by a firm blow to the head, followed by severing of the spinal cord with a scalpel. The eyes were gently removed using a curette made from a sharpened spatula and fine surgical scissors. Vitreous fluid was removed from some eyes using a plastic micropipette tip inserted gently through the optic disc. Glycerol concentrations were determined using a diagnostic kit (33740A; Sigma-Aldrich, Oakville, ON, Canada) and following the manufacturer’s instructions. Thermal hysteresis caused by the non-colligative antifreeze activity was measured as the difference between melting and freezing points using a Clifton Nanolitre Osmometer (Clifton Technical Physics, Hartford, NY). Antifreeze proteins decrease the freeze point of a solution but do not influence the melt point. This difference calculated in osmotic pressure is referred to as thermal hysteresis. Thermal hysteresis was calculated as (freeze point °C- melt point °C) times (1.86 °C/1000 mOsmols) based on the fundamental physical property that the freezing temperature of a solution with an osmotic pressure of 1000 mOsmols is −1.86 °C [1].

Eyes that were not used for vitreous fluid collection were snap frozen or paraffin embedded for tissue and protein expression analyses by histology, western blot, and immunohistochemistry. For histology specimens, paraffin sections were prepared, registered, and stored in serial or adjacent order. Every twentieth paraffin section was stained with hematoxylin and eosin (H&E) to map retinal structures such as the rete mirabile and its junction with the choriocapillaris.

For western blots of smelt retinal tissues, pieces of smelt retinas were carefully dissected from snap frozen smelt eyes using a scalpel blade in the frozen chamber of a cryostat. Retinal tissues were homogenized in a lysis buffer (50 mM Tris-HCl pH 7.6, 150 mM NaCl, and 1% Triton X-100) supplemented with protease inhibitors (1 mM phenylmethylsulfonyl fluoride, 0.3 U/ml aprotinin, and 10 μg/ul leupeptin) and phosphatase inhibitors (1 mM sodium orthovanadate, 25 mM sodium fluoride, and 10 mM β-glycerophosphate). Lysates were clarified by centrifugation and proteins were quantified. Western blots for Tbdn and ZO-1 were performed by standard procedures using a LumiGLO reserve chemiluminescence substrate kit (Mandel Scientific, Guelph, Ontario, Canada). Tbdn western blots were performed using affinity purified anti-Tbdn antibody C10–20 raised in rabbit, against Tbdn C10–20 peptide corresponding to the amino acid sequence EAWTKYPRGL of mouse Tbdn [14]. For blocking peptide experiments, western blots were performed in the presence of 200 μM of Tbdn C10–20 blocking peptide EAWTKYPRGL or 200 μM of a control peptide corresponding to another region of mouse Tbdn protein (amino acid 755 to 766). For the ZO-1 western blot analysis, mouse monoclonal anti-human ZO-1 1A12 (Zymed, Carlsbad, CA) was used. Tbdn and ZO-1 western blots were stripped and re-probed with an anti-α-Tubulin mouse monoclonal antibody (Sigma, St. Louis, MO) to verify protein integrity and loading equivalency.

Tbdn expression was also analyzed by immunohistochemistry using the previously described OE5 mouse monoclonal anti-Tbdn antibody raised against the same peptide as the anti-Tbdn C10–20 antibody, corresponding to the amino acid sequence EAWTKYPRGL of mouse Tbdn [23]. For immunostaining, sections from paraffin embedded tissues were deparaffinized, post-fixed in 4% paraformaldehyde, and incubated overnight with a primary antibody or a negative control isotype match IgG2a antibody (DakoCytomation, Glostrup, DK) in 3% fat-free powdered skimmed milk in TBS with 0.05% Tween-20 (TBST). Sections were developed using the appropriate alkaline phosphatase conjugated secondary antibodies (anti-mouse IgG or anti-mouse IgG2a) and a Vector Red substrate kit (Vector Laboratories, Burlingame, CA). Sections were then air dried and mounted in Permount (Fisher Scientific, Pittsburgh, PA).

For ZO-1 immunohistochemistry, antigen retrieval was performed by incubating the sections with trypsin (1 mg/ml, 0.1% w/v in 150 mM Tris, pH 7.6, 3.3 mM calcium chloride; Immunon, Pittsburgh, PA) for 10 min at 37 °C, followed by washing for 5 min in TBS. Blocking was performed with a 2% ECL Advanced Block (GE Healthcare, Baie d’Urfe, QC, Canada) in TBS for 1 h to block nonspecific binding sites. After blocking, the sections were incubated with the primary antibody, mouse monoclonal anti-ZO-1 1A12 (Zymed), at 1:100 dilution at room temperature overnight. The sections were then washed with TBS three times for 5 min each, followed by incubation with anti-mouse IgG (H^+^L) alkaline phosphatase (AP) conjugate (Promega, Madison, WI) for 1 h. The AP activity was detected using an AP substrate kit as described above.

Immunohistochemical reactions were quantified by measuring staining intensities as previously described [14]. Briefly, sections were viewed and photographed using a Leica (Bannockburn, IL**)** DMIRE2 microscope system equipped with a QImaging (Surrey, BC, Canada) RETIGA Exi camera and with Improvision (Coventry, UK) Openlab (Version 5) software. The intensity of Tbdn and ZO-1 staining in blood vessels was measured by determining the intensity of the color red using the HIS Colorspy tool from Openlab software. Tbdn and ZO-1 levels in the rete mirabile, choroidal blood vessels, and other areas of the eye were expressed as the average staining levels of at least three separate specimens. Relative intensities were expressed as the average staining levels±the standard error of the mean (SEM).

Values were expressed as the mean percentage of the control±SEM. Quantitative analyses were compared using the two-tailed Student's *t* test. The data were considered to be statistically significant if the p-value was less than or equal to 0.05.

## Results and Discussion

**Figure 1:**
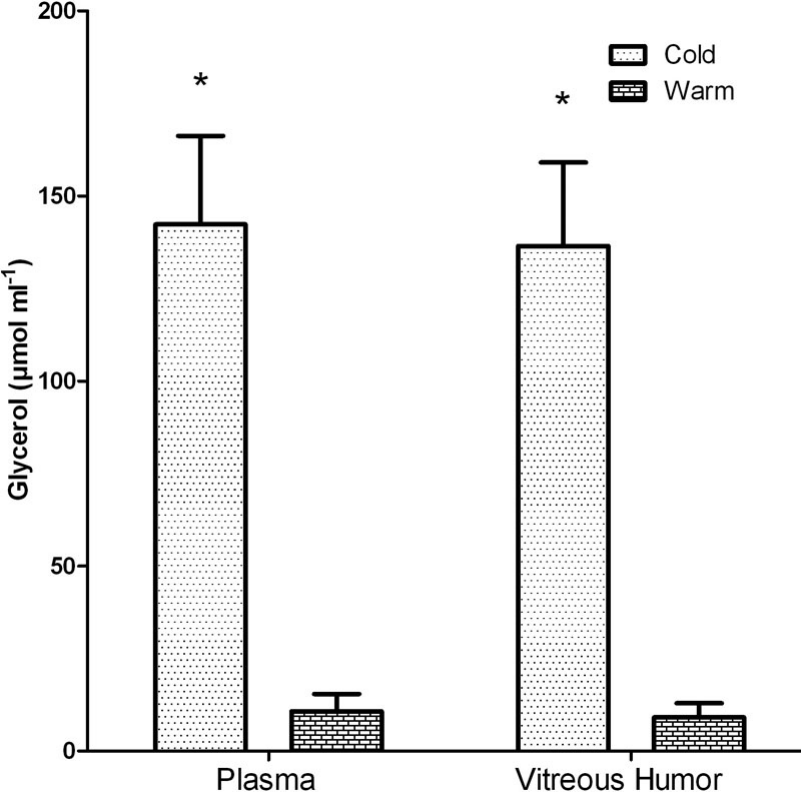
Glycerol levels in the plasma and vitreous fluid of smelt at warm (8–10 °C) and cold (<0.5 °C) temperatures. The values are presented as mean±SEM, with n=5 in all groups. * indicates a statistically significant difference.

The level of glycerol in the vitreous fluid was significantly higher in fish living in cold versus warm temperatures. For fish sampled in January 2008, the glycerol level in vitreous fluid was 153±21 (n=6) mM in cold fish and 4.36±4.16 mM (n=5; four values under 0.34 mM and one value of 21 mM) in warm fish. This is the first report of the accumulation of glycerol in the vitreous fluid of freeze-resistant smelt. This phenomenon was confirmed in fish sampled in later years, where the glycerol level was 136 and 9.2 mM, in cold and warm fish, respectively (Figure 1). This experiment also reports the level of glycerol in cold and warm fish plasma as being 142 and 10.1 mM, respectively, revealing that there is an equilibration of glycerol between the plasma and vitreous fluid.

**Figure 2:**
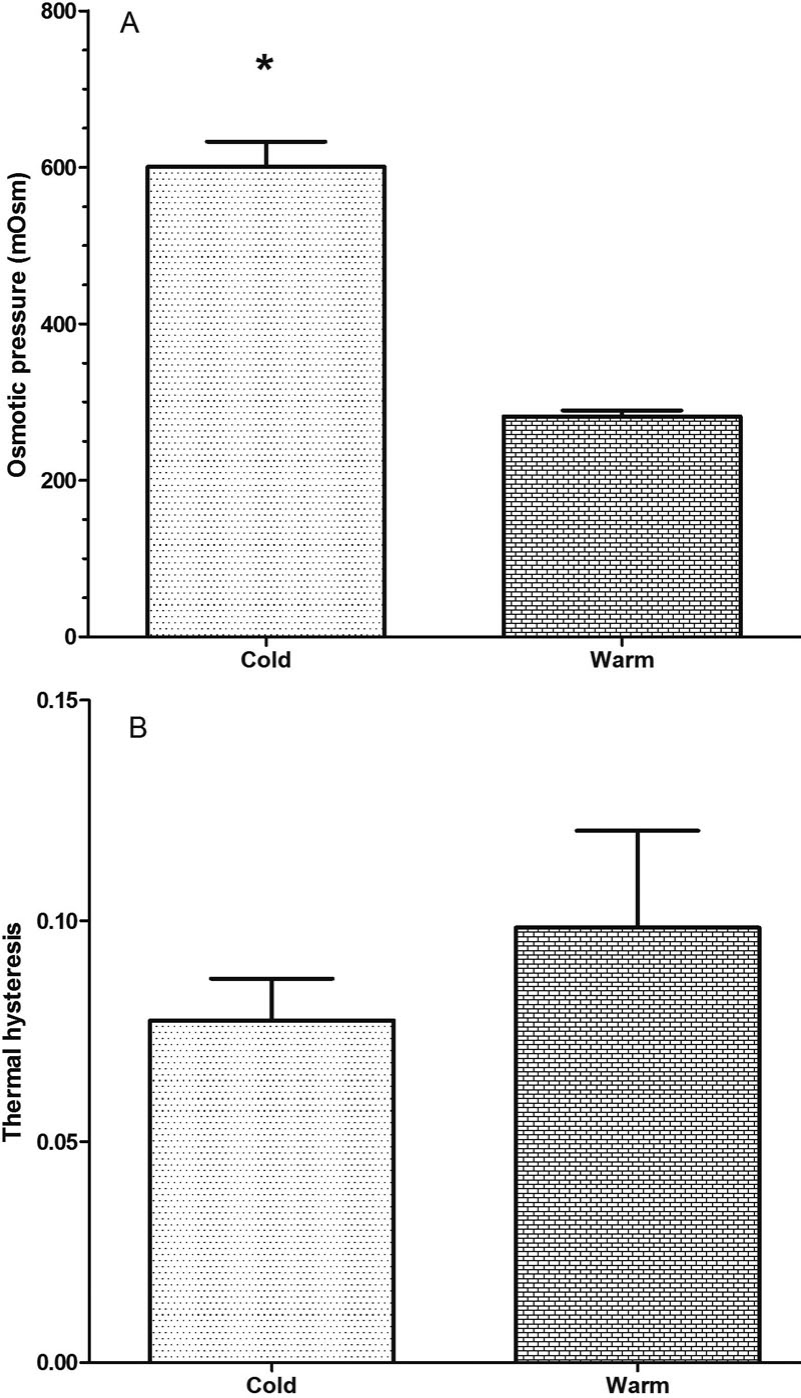
Osmotic pressure in vitreous fluid in cold versus warm fish. **A**: Osmotic pressure in vitreous fluid in smelt at warm (8–10 °C) and cold (<0.5 °C) temperatures. **B**: Thermal hysteresis in vitreous fluid in smelt at warm (8–10 °C) and cold (<0.5 °C) temperatures. The values are presented as mean±SEM with n=3 for the warm group and n=5 for the cold group. * indicates a statistically significant difference.

The osmotic pressure in vitreous fluid was significantly higher in cold versus warm fish—being twofold higher at cold versus warm temperatures (Figure 2A). The freezing point of the vitreous humor (calculated from the osmotic pressure) was −1.20±0.9 °C and −0.62±0.03 °C for cold and warm smelt, respectively. The freeze point depression was sufficient to prevent freezing in ambient water temperatures during winter. The difference in osmotic pressure of about 300 mOsmols could not be fully accounted for by glycerol accumulation, which was only about 125 mM higher in cold compared to warm fish. This implies that other osmolytes accumulate in the vitreous fluid as well as glycerol. These results indicate that, like the rest of a fish's body, a hyperosmotic physiologic adaptation—partly involving glycerol accumulation—protects the rainbow smelt eye from freezing in subzero water temperatures.

The level of thermal hysteresis was similar and very low in the vitreous fluid of both cold and warm smelt (Figure 2B). The contribution of any freeze resistance in vitreous fluid due to the presence of antifreeze proteins was likely less than 0.1 °C. Although not measured in this study, the level of thermal hysteresis in plasma at sampling dates has been reported to be between 0.25 °C and 0.5 °C [3,22]. This implies that there is a barrier to the movement of the antifreeze protein from the plasma space into the vitreous fluid. This conclusion is similar to findings from Antarctic fish, where the measured level of glycopeptide antifreeze is much higher in serum than in aqueous humor [21].

**Figure 3:**
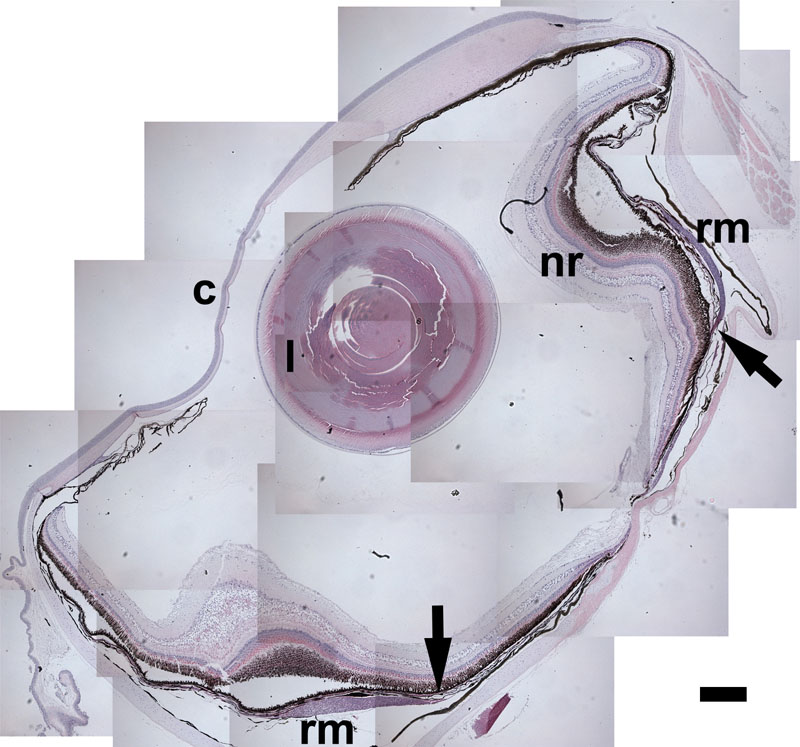
A transverse section of a whole smelt eye showing the cornea (c), lens (l), neural retina (nr), and rete mirabile (rm). The arrows indicate the junction between the rete mirabile and the choriocapillaris. Hematoxylin and Eosin, 50×. The composite image was created using tiling of multiple frames to capture the entire globe at a high resolution. The scale bar in the lower right corner of the figure represents 400 μm.

Studies have yet to assess if hyperosmotic adaptation, which offers smelt protection from freezing, affects cellular permeability pathways that could impact molecular traffic in the eye. We sought to explore this by applying knowledge gained on retinal endothelial permeability pathways in mammalian systems to the smelt. A low power photomicrograph of the key elements in the smelt eye is presented in Figure 3. The smelt eye is similar to those reported in other teleost species [5,6] and includes the presence of a rete mirabile, the circulation of which is continuous with that of the choriocapillaris.

**Figure 4:**
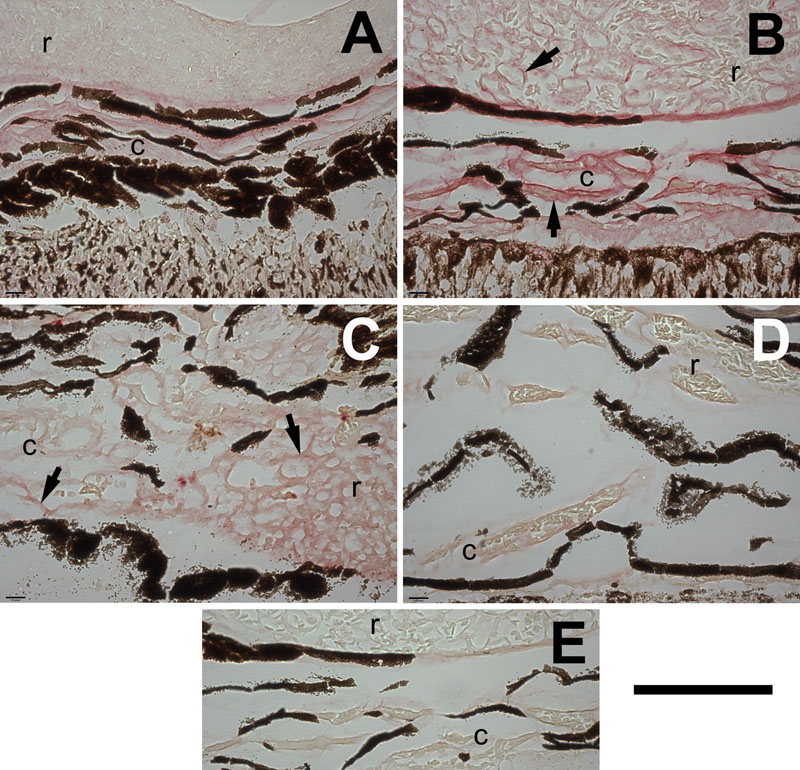
Tbdn and ZO-1 proteins show reciprocal regulation in cold-adapted smelt rete and choroidal blood vessels. Compared to warm fish maintained at 8–10 °C (**A**: warm specimen/Tbdn stain; **C**: warm specimen/ZO-1 stain), the endothelial linings of the choroidal (c) and rete (r) blood vessels of cold fish maintained at 0.5 °C show a higher expression level of Tbdn protein, but a lower level of ZO-1 protein in these regions (**B**: cold specimen/Tbdn stain; **D**: cold specimen/ZO-1 stain). The arrows indicate choroidal and rete blood vessel endothelia. **E**: Sections were also incubated with a control IgG and showed no staining of the blood vessels. Positive staining for Tbdn (OE5) and ZO-1 appears as bright red staining. The dark brown or black color in all panels is intrinsic due to pigmentation from the pigments cells of the choroidal vasculature and/or retinal tissue. The immunohistochemical results shown here are representative of three smelt in each of the warm and cold groups and are quantitated in Figure 6. The scale bar in the lower right corner of the figure indicates 100 μm.

**Figure 5:**
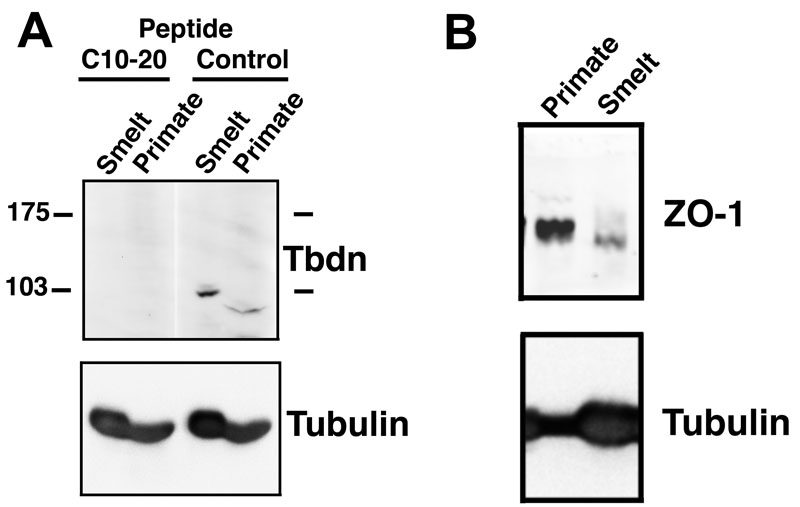
Western blot analyses of smelt orthologs of mammalian Tbdn and ZO-1 proteins. **A**: A Tbdn western blot analysis showing a major band of approximately 103 kDa in smelt and 100 kDa in primate cells reacting with affinity purified rabbit anti-mouse Tbdn C10–20 antibody, which are completely competed out by the addition of the C10–20 Tbdn peptide, but not by a control peptide, as indicated. The bottom panel shows Tubulin as a protein loading and integrity control. **B**: A ZO-1 western blot analysis showing a doublet in primate and smelt cells reacting with the anti-ZO-1 antibody, as indicated. The bottom panel shows Tubulin as a protein loading and integrity control.

A teleost ortholog of the mouse *Tbdn* gene, the protein product of which represents a marker of the endothelial transcellular permeability pathway, exists [National Center for Biotechnology Information resources: Expressed sequence tags (EST): GE781036.1, EG915740.1, DY704791.1, DY734706.1, CX355128.1, and CX066490.1]. In addition, the mouse Tbdn peptide epitope (C10–20) against which we raised a Tbdn specific monoclonal antibody reagent (OE5) displays approximately 90% homology with salmonid and osmerus mordax expressed sequence tags (EST: CX355128.1, CX066490.1, EL547336 and EL536341.1). Moreover, our anti-Tbdn monoclonal antibody (OE5) stained retinal choriocapillaris and rete mirabile blood vessels in smelt eyes, and these have similar anatomic structures to those in which we observed Tbdn immunostaining in mammals [9–12] (Figure 4). To further confirm the specificity of mouse Tbdn peptide epitope C10–20 and the OE5 monoclonal antibody for a putative smelt Tbdn protein, western blot analyses on smelt retinal tissues were performed. Since our Tbdn monoclonal antibody OE5 is not useful for western blotting applications, our affinity purified rabbit anti-Tbdn antibody C10–20, which is raised against the same epitope as our OE5 antibody, was used. A western blot analysis of smelt retinal tissue using the anti-Tbdn antibody C10–20 revealed a major band of ~103 kDa (Figure 5). Moreover, the reactivity of the C10–20 Tbdn antibody with the 103 kDa protein present in smelt retinal tissues was competed away by the presence of the competing peptide Tbdn C10–20 as compared to a control peptide (Figure 5). Similar results were obtained with primate retinal endothelial cell line protein extracts (Figure 5). This data strongly suggests that smelt harbor a Tbdn ortholog. The slight difference in molecular weight between the smelt (103 kDa) and primate (100 kDa) Tbdn proteins might reflect their evolutionary divergence. Additional studies are required to clarify these differences. However, this is the first report of a putative smelt ortholog for the mammalian Tbdn protein.

We found evidence in National Center for Biotechnology Information resources for a legitimate mature transcript of the teleost ZO-1 gene in two teleost species, oncorhynchus mykiss and osmersu mordax. These teleost ZO-1 homologous expressed sequences are represented in BX303254.3 and EL549507.1, respectively. The epitope harbored within the predictable protein product, against which commercially available anti-ZO-1 antibody reagents were raised, displays a range of 50%–80% identity between smelt and mammalian sequences. We found that the 1A12 mouse anti-human ZO-1 antibody (Zymed), which is also reactive against mouse and dog ZO-1, stained several ocular structures that were consistent with the previously described immunolocalization of ZO-1 protein in mammalian eyes [24]. These structures include the outer limiting membrane of the neural retina, the corneal endothelium, and iris blood vessels (not shown). In addition, western blot analyses using the anti-ZO-1 antibody 1A12 revealed a doublet in smelt retinal tissues co-migrating with the mammalian ZO-1 protein doublet present in a primate retinal endothelial cell line RF/6A [14] (Figure 4). In addition, we found that the mouse anti-ZO-1 antibody also stains the endothelial linings of the rete mirabile and the blood vessels with which the rete merges with the choriocapillaris at its junction (as indicated in Figure 3). These results suggest that the bands with which antibody reagents raised against mouse Tbdn and human ZO-1 react in western blots of smelt retinal tissue are smelt orthologs of the corresponding mammalian counterparts. Since the Tbdn and ZO-1 epitopes can also be detected by immunohistochemistry in similar specific anatomic structures in both mammals and smelt eyes, our results suggest that the antibody reagents we tested are useful for detecting smelt Tbdn and ZO-1 proteins in smelt retinal tissues. These reagents were thus used to examine the pattern of expression of these two markers of endothelial permeability in warm- and cold-adapted specimens.

**Figure 6:**
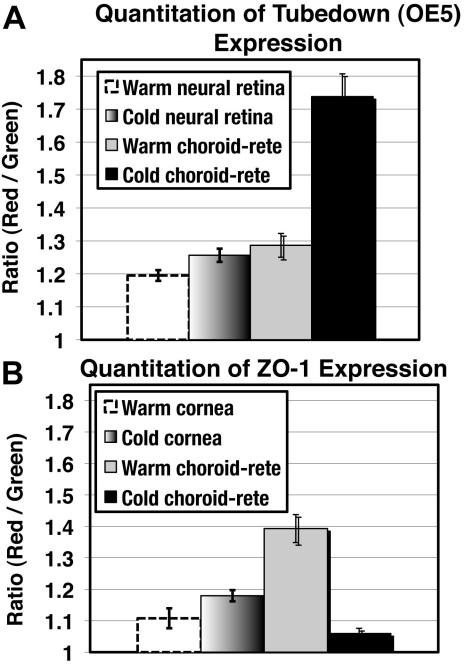
Quantitation of staining by a mouse anti-Tbdn antibody (**A**) and by a mouse anti-ZO-1 antibody (**B**) on smelt eye tissues. Compared to fish maintained at 8–10 °C (warm), there was a significant increase (p<0.001; n=15 color intensity measurements per group) in Tbdn staining of rete-choroidal blood vessels in the cold-adapted smelt (maintained at 0.5 °C [cold]), as shown in panel **A**. There was no significant difference in the staining of Tbdn in the neural retina. Compared to fish maintained at 8–10 °C (warm), there was a significant decrease (p<0.001; n=15 color intensity measurements per group) in ZO-1 staining of rete- choroidal blood vessels in the cold-adapted smelt (maintained at 0.5 °C [cold]), as shown in panel **B**. There was no significant difference in the staining of ZO-1 in the cornea.

Compared to three individual warm-adapted specimens, the endothelial linings of the choroidal and rete mirabile blood vessels of three individual cold-adapted specimens showed a higher expression level of Tbdn in these regions (Figure 4). Interestingly, expression of the zonula occludens protein ZO-1 showed an expression pattern reciprocal to that displayed by Tbdn. ZO-1 was downregulated in the endothelial linings of the choroidal and rete mirabile blood vessels of three individual cold-adapted specimens compared to three individual warm-adapted specimens (Figure 4). Expression levels of Tbdn and ZO-1 in neural retina and cornea, respectively, were not significantly different between warm and cold-adapted specimens (see quantitation in Figure 6). This data serves as an internal positive control and supports the validity of the temperature-related changes in Tbdn and ZO-1 in the choroid-rete tissues. While it was possible to detect low levels of what we believed to be smelt orthologs of mammalian Tbdn and ZO-1 proteins in smelt whole retinal tissue lysates (Figure 5), it was not possible to dissect out sufficient amounts of tissue from the choroid-rete junction to accurately perform comparisons of Tbdn and ZO-1 protein expression in temperature-adapted fish by western blot. However, western blot detection of the putative smelt homologs using antibodies raised against the same mouse epitopes supports validation of the immunohistochemical results.

The hyperosmotic physiologic adaptation involving glycerol, which protects the smelt eye from freezing in subzero water, suggests that a molecular mechanism exists in the vasculature of a smelt eye to manage the delivery (and perhaps the consequences) of glycerol and other osmolytes to the vitreous humor. Since Tbdn is associated with a transcellular endothelial permeability pathway, while ZO-1 is associated with a paracellular endotheial permeability pathway, the reciprocal expression pattern of these proteins suggests that cold adaptation in smelt eyes might involve the adaptation of two different permeability pathways in the blood vessels of the rete mirabile and choriocapillaris, which manages the movement of antifreeze materials into the eye. The rete mirabile and the choriocapillaris are essential to the delivery of oxygen and nutrients to the retina of teleosts [5,6]. In the mammal, the choriocapillaris is fenestrated, but the roles of the endothelial transcellular permeability and paracellular permeability pathways in choroidal vascular homeostasis are not well understood. Regardless of the state of fenestration of these vasculatures, our results might suggest that the permeability of the rete-choriocapillaris vasculatures is challenged by hyperosmotic stress and adapts accordingly through adjustments of the two major endothelial permeability pathways.

The mechanisms underlying these changes and the functions affected remain unexplored. However, since Tbdn seems to help limit the transcellular passage of Albumin in normal mammalian retina [14], one might hypothesize that the upregulation of Tbdn protein expression in cold stressed smelt might involve a signaling pathway that regulates the passage of antifreeze proteins into the retina, which, like extravascular Albumin leakage in mammalian retinopathy, could be detrimental to the retinal tissues. Likewise, since ZO-1 protein is a zonula occludens molecule, the downregulation of ZO-1 protein expression in cold stressed smelt might allow small antifreeze molecules—such as glycerol—through paracellular passages to provide readily available plasma-based antifreeze to the retina and vitreous.

This work presents the first evidence that molecules known to play a role in ocular vascular homeostasis may be differentially regulated during hyperosmotic anti-freezing cold adaptation in smelt eyes. These results provide fundamental insight into the physiologic adaptation to internally generated osmotic change in smelt. Local intraocular microenvironmental osmotic stress due to hyperglycemia of diabetes in mammals leads to advanced glycation end products which correlate with microvascular retinal lesions [25,26]. The nature of how retinal pathology is avoided in the adaptation of the smelt eye to osmotic stress could provide valuable new insights that may be useful for understanding the pathobiology of diabetic vasculopathy in mammals. Recent work indicates that changes in the transport of glycerol across the blood-retinal barrier have direct relevance to diabetic retinopathy [27]. Therefore, this work might further our fundamental understanding of the mechanisms underlying adaptation of the blood-retinal barrier to metabolically relevant compounds such as glycerol.
